# Improvements in the Procedures to Encapsulate Diverse Bioactive Compounds

**DOI:** 10.3390/foods11020205

**Published:** 2022-01-12

**Authors:** Teresa Antequera, Juan Carlos Solomando, Trinidad Pérez-Palacios

**Affiliations:** Research Institute of Meat and Meat Products (IProCar), University of Extremadura, Avda. de las Ciencias s/n, 10003 Caceres, Spain; tantero@unex.es (T.A.); jsoloman@alumnos.unex.es (J.C.S.)

Published articles within the “Microencapsulation of Bioactive Compounds: Techniques and Applications” special issue have been mainly focused on the evaluation of variables affecting the encapsulation of healthy compounds, aiming to achieve accurate quality properties in the encapsulates. Most publications have analyzed variables related to the encapsulation process, applying different techniques and carrying out several determinations to evaluate the quality of the encapsulates themselves. There are few articles at evaluating the influence of the addition of the encapsulates to food.

Five main groups of publications can be differentiated depending on the bioactive compounds they have focused on: with recognized antioxidant properties (tomato oil as a source of lycopene, thyme essential oil as a source of phenolic compounds, oleoresin of Haematococcus pluvialis as a source of astaxanthin, Cornelian cherry fruits as a source of anthocyanins and resveratrol solutions), with high content in polyunsaturated fatty acids (hempseed oil and fish oil as source of omega-3 fatty acids), flavones (tangerine oil as a source of tangeretin and a commercial genistein solution), flavorings (ethyl acetate and vanilla) and probiotics (*Lactobacillus rhamnosus* and *L. casei*).

Table 1 summarizes the encapsulation variables evaluated for the different bioactive compounds that have been studied in the publication of this issue. Starting with those compounds with recognized antioxidant properties, the ability of α-, β- and γ-cyclodextrins to stabilize emulsions with tomato oil and to form powders has been evaluated by Durante et al. [1]. α-cyclodextrins achieved the highest encapsulation efficiency, while β-cyclodextrins showed the best oil dispersion. α- and β-cyclodextrins demonstrated better performance than γ-cyclodextrins in relation to the antioxidant activity of emulsions and powders, although their stability was not affected by the cyclodextrin type, with a rapid decline in the carotenoid content. On the other hand, the capability of γ-cyclodextrins to form a complex with resveratrol has been confirmed by Silva et al. [2], who showed a true inclusion complex with the resveratrol inside the cavity of the γ-cyclodextrin channels. In the study of González-Reza et al. [3], the influence of different encapsulating (poly-ε-caprolactone and ethylcellulose) and stabilizing (polyvinyl alcohol and Pluronic^®^ F-127) polymers on quality parameters of thyme essential oil nanocapsules has been evaluated. The use of poly-ε-caprolactone and polyvinyl alcohol had a positive effect on physical, antioxidant and stability properties of the nanocapsules of thyme essential oil, being inferred its potential application in food processing and preservation. The effect of including dodecanol as a membrane stabilized in resveratrol niosomes formulated with mixtures of Tween 80 and Span 80 has been evaluated by Machado et al. [4]. The presence of dodecanol preserved the antioxidant capacity and release of resveratrol without affecting the general properties of niosomes, such as size or shape. The use of lupin protein isolate, carrageenan and chitosan as ionic interfacial layers to stabilize multilayer emulsions of astaxanthin oleoresin has been evaluated by Morales et al. [5]. In this study, lupin protein isolate has been used to obtain the first layer, which covers the oily phase. Then, carrageenan and chitosan were added over, obtaining second and third layers, respectively. Although all emulsions stabilized with one, two, and three interfacial layers presented greater physical stability, those with two layers were the most stable when subjected to different pHs, and higher astaxanthin retention was found with two and three layers in comparison to one. Thus, stable multilayer emulsions with astaxanthin microencapsulated can be produced using lupin protein isolate, carrageenan and chitosan as ionic interfacial layers. Dumitrascu et al. [6] have investigated the influence of pasteurization and sterilization treatments on the potential of soy proteins to ensure the efficient encapsulation and gastric stability of anthocyanins from cornelian cherry fruits, based on previous results that demonstrated the influence of the wall material on the encapsulation of these bioactive compounds. Preheated soy proteins reduced the encapsulation efficiency but improved the stability of anthocyanins during gastric digestion. Thus, the authors of this work considered it necessary to identify the optimum heat conditions for soy proteins to allow higher encapsulation efficiency of the target compounds.

As for oils rich in polyunsaturated fatty acids, the use of a combination of maltodextrin with hemp, pea or rice proteins, as coating materials, to encapsulate hempseed oil has been studied by Kurek et al. [7]. These authors have also investigated the effect of the level of oil loaded. The two evaluated variables significantly influenced the encapsulate characteristics. Both rice and pea proteins were found to be a viable plant-based alternative for wall material in terms of encapsulation efficiency, and the use of 10% oil loading and the combination of maltodextrin with pea protein achieved the best results. Solomando et al. [8] have evaluated fish oil microcapsules from emulsions with different interfacial layers, lecithin + maltodextrine (monolayered) and lecithin + maltodextrine + chitosan (multilayered). These authors focused on the profile of volatile compounds of the powders, finding a low abundance of volatile compound from lipid oxidation in monolayered microcapsules. This may indicate the major protection of the maltodextrine-chitosan layer against fish oil oxidation.

Some authors have also focused on the encapsulation of flavones in this special issue. Xiao et al. [10] have prepared two types of nanoparticles (with zein and zein/carboxymethyl chitosan as covering materials) of genistein. The encapsulation of genistein in zein/carboxymethyl chitosan better improved water dispersibility, antioxidant activity, stability and controlled released. In the research of Sun et al. [11], an oil-in-water emulsion of bergamot oil with tangeretin has been developed, using sodium alginate and pectin as wall material, and evaluating the percentage of oil loaded. The combination of pectin and sodium improved stability and encapsulation efficiency, due to the increased incorporation and concentration of crosslinked polymers in the final matrix. The main findings of this study demonstrated the capability of different encapsulated concentrations of tangeretin in a pectin/sodium alginate matrix using bergamot oil as a carrier. Nevertheless, the retention efficiency increased and the encapsulation efficiency decreased as the concentration of tangeretin rose.

The encapsulation of flavorings has also been aimed among the published investigations of this issue. Hernández-Fernández et al. [12] have developed vanilla oleoresin microcapsules, evaluating the conditions of complex coacervation (percentage of gum Arabic and chitosan, and pH) and the influence of spray-drying. According to the findings of this work, it is possible to microencapsulate vanilla oleoresin extracted through complex coacervation and spray-drying, which allowed the protection of the volatile compounds to be increased and a higher stability. The optimal conditions found were 0.34% and 1.7% of gum Arabic and chitosan, respectively, at pH 5.29. Li et al. [12] have investigated the effect of maltodextrine with different dextrose equivalent values (6, 7 and 12) to encapsulate ethyl acetate. In this study, the use of maltodextrine with a high dextrose equivalent, and whey protein as wall materials provided the best protection for flavor substances during storage, and increased their release during dissolution.

In this issue, some authors have developed new coating material systems of microencapsulation to protect probiotic. Sun et al. [15] have modified the charge of a pectin methylesterase extracted from citrus pulp, which was used to encapsulate *L. casei* by ionotropic gelation. These authors have also evaluated the storage conditions of the hydrogels, at 4 °C or freeze-dried. The charge modified pectin shows a high encapsulation efficiency and stability under gastrointestinal simulations, especially when freeze-dried. Oberoi et al. [14] encapsulated *L. rhamnosus* into alginate microbeads that were coated with different materials (xanthan gum, gum acacia, sodium caseinate, chitosan, starch, carrageenan). The alginate + xanthan gum formulation showed a significantly higher encapsulation efficiency (95%) than other coating agents and increased the survival rate of the cells in stress conditions during gastrointestinal transition compared to the free bacteria.

Some studies of this issue have also dealt with the effects of additional encapsulates of bioactive compounds to food. Silva et al. [2] aimed to investigate the impact of including a resveratrol complex as a functional ingredient to lemon juice, concluding that a complex made with maltodextrine promotes the solubility and antioxidant stability of reveratrol in lemon juices. In the studies of Solomando et al. [8,9], who enriched the omega-3 fatty acids in different meat products (cooked and dry-cured sausages) by adding two types of fish oil microcapsules (monolayered and multilayered), the abundance of volatile compounds from lipid oxidation and markers of omega-3 oxidation was higher when adding monolayered microcapsules. Moreover, findings indicated that the changes in volatile compounds during storage depend on the type of fish oil microcapsules and the meat products, having an increased abundance of omega-3 oxidation markers in dry-cured sausages added with monolayered microcapsules. However, the enrichment of cooked sausages with fish oil microcapsules did not modify the usual variations in the volatile compound profile during culinary cooking. Thus, it seems that a multilayered structure of chitosan–maltodextrin may achieve greater fish oil protection than the simple coating of maltodextrin in microcapsules.

Regarding the encapsulation techniques used in the works published in this special issue, spray-drying has been the most applied one. It has been used to form microcapsules of astaxanthin [5], hempseed oil [7], fish oil [8,9], tangereting [10] and vanilla [11] from emulsions previously prepared. For astaxanthin and tangereting, the oil phase of emulsions was prepared with sunflower and bergamot oil, respectively [5,15], whereas an oleoresin extraction was carried out in the case of vanilla [11]. In these studies, a mini spray-dryer has been used, applying similar conditions (inlet temperature 100–180 °C, feed rate 3–16 mL/min, aspiration 80–100%). The freeze-drying method has been applied to encapsulate sources of bioactive compounds with antioxidant recognized properties, tomato oil [1], resveratrol [2] and anthocyanins [6]. An emulsion of tomato oil and solutions of resveratrol and anthocyanins have been freeze-dried in these works to obtain the corresponding encapsulates, namely powders, inclusion complexes and microcapsules, respectively. In the case of probiotics, both studies of this issue have applied a gelation procedure to encapsulate the bacteria with sodium alginate or pectin and calcium chloride [14,15]. Nanocapsules of genistein [9] and thyme essential oil [3] have been prepared by a phase separation procedure with a final lyophlization, and an emulsification–diffusion method with ultra-high agitation, respectively. The thin film hydration method has been also applied to prepare niosomes of resveratrol by evaporating the solvents inside a vacuum [4]. Besides, in this issue, a review on supercritical carbon dioxide techniques (Particles from Gas Saturated Solutions, Particles from Gas Saturated Solutions Drying, Rapid Expansion of Supercritical Solutions, Gas Anti-Solvent, Supercritical Anti-Solvent Process, Solution Enhanced Dispersion by Supercritical Fluid, Supercritical Fluid Extraction of Emulsions) to encapsulate bioactive compounds has been published [16]. Although different types of encapsulated bioactive compounds can be produced by means of these supercritical carbon dioxide techniques, there are some drawbacks that need to be considered: the solubility of the active compounds in the carrier material, which limit the concentration of the encapsulated compound; solvents used are not food grade; most used carrier materials are synthetic polymers; lack of knowledge about the physicochemical and functional properties of the final powders. Thus, more studies are required to optimize the processing variables and understand the characteristics of the obtained encapsulates.

In the studies of this special issue, the encapsulates have been preferentially analyzed by means of the Fourier Transform Infrared Spectroscopy (FTIR), Scanning Electron Microscopy (SEM), particle size distribution, Polydispersity Index (PDI), Zeta-Potential, density, moisture content, encapsulation efficiency, stability, release and in vitro digestion. In the FTIR analysis, the absorption spectra of samples ranged between 4000 and 400 cm^−1^ with a resolution of 4 cm^−1^, allowing the solid characterization of the encapsulates. Particle size, PDI, Zeta-potential are normally determined by a dynamic scattering of light, using a Zetasizer Nano [9,11]. PDI is a measure of the width of the particle size distribution and Zeta-potential gives information about possible interactions among compounds in the encapsulates. Bulk and tapped density can be determined to measure the flow properties of the powders, which can impact on transportation, packaging and mixing. High bulk density values suppose small container requirements and the amount of air that decreases the susceptibility to oxidation. Moisture percentage is determined by the gravimetric method, based on drying in an oven at temperatures higher than 102 °C until at a constant weight. Encapsulation efficiency has been determined in most of the published papers of this issue, mainly as the final aim is to increase it as much as possible. It is estimated based on the content of the bioactive compound that is encapsulated in relation to the total amount in the encapsulate. The stability of the bioactive compounds at different conditions has also been evaluated. The content of carotenoids in tomato oil microcapsules stored at different temperatures and times was determined by Durante et al. [1], and a similar assay was conducted by Xiao et al. [9] in nanoparticles of genistein. Morales et al. [5] quantified the concentration of resveratrol in niosomes under ultraviolet light. The effect of room and refrigeration temperatures on the survival rate of *L. casei* hydrogels has been analyzed by [13]. The release of encapsulated bioactive compounds has also been determined by means of different conditions and methods. In the case of the thyme essential oil nanocapsules, they were suspended in clyclohexane and ultracentrifugated, quantifying the carotenoids released over time [1], while Kurek et al. [7] used an aqueous model (phosphate buffer solution) to analyze the release of hempseed oil from microcapsules. The flavor release of ethyl acetate microcapsules was investigated under different relative humidity, to imitate storage environments [13], and the dialysis membrane method was used to achieve the release of genistein from nanoparticles [13]. Moreover, some of the encapsulates of these studies have been subjected to simulated gastrointestinal digestion.

The following conclusions can be drawn from publications within the “Microencapsulation of Bioactive Compounds: Techniques and Applications” especial issue: the great interest in improving encapsulation conditions to obtain accurate encapsulates of bioactive compounds, especially in the search of optimum wall materials; the need for encapsulation diverse bioactive compounds (with recognized antioxidant properties; omega-3 fatty acids; flavons; flavorings; probiotics); spray-drying goes on being the most used technique to encapsulate bioactive compounds; the aim of increasing encapsulation efficiency and stability during storage and gastrointestinal digestion; getting knowledge about structure and physical properties of the encapsulates.

## Figures and Tables

**Table 1:** Evaluated variables for encapsulation different bioactive compounds in the “Microencapsulation of Bioactive Compounds: Techniques and Applications” especial issue.

Main Property	Encapsulated Material-Compounds	Encapsulation Variable	Reference
Antioxidant	Tomato oil-carotenoids	Type of wall material: α-, β- and γ-cyclodextrins.	[1]
	Thyme essential oil-phenolic compounds	Different encapsulating (poly-ε-caprolactone and ethylcellulose) and stabilizing (polyvinyl alcohol and Pluronic^®^ F-127) polymers.	[3]
	Resveratrol solution	Use of γ-cyclodextrins.	[2]
	Resveratrol solution	Use of dodecanol as membrane stabilizer.	[4]
	Astaxanthin oleoresin	Use of lupin protein isolate, carrageenan and chitosan as ionic interfacial layers to stabilize multilayer emulsions.	[5]
	Cornelian Cherry fruits-anthocyanins	Effect of temperature on the potential of soy proteins as wall material	[6]
Rich in PUFA	Hempseed oil	Percentage of oil loaded. Use of maltodextrine in combination to different plant-based proteins (hempseed, pea and rice), as wall material.	[7]
	Fish oil	Different interfacial layers: lecithin+maltodextrine vs. lecithin+maltodextrine+chitosan.	[8,9]
Flavons	Genistein solution	Zein vs. zein/carboxymethyl chitosan as wall material.	[10]
	Tangerine oil-tangeretin	Percentage of oil loaded in an oil-in-water of sodium alginate and pectin as wall materials.	[11]
Flavorings	Vanilla	Conditions of complex coacervation conditions (percentage of gum Arabica and chitosan, and pH). Influence of spray-drying after complex coacervation.	[12]
	Ethyl acetate	Maltodextrine with different dextrose equivalent.	[13]
Probiotics	*L. rhamnosus*	Alginate microbeads coated with different materials (xanthan gum, gum acacia, sodium caseinate, chitosan, starch, carrageenan).	[14]
	*L. casei*	Use of modified charge pectin. Cooling or freeze-drying hydrogels.	[15]
